# Development of SARS-CoV-2 packaged RNA reference material for nucleic acid testing

**DOI:** 10.1007/s00216-021-03846-y

**Published:** 2021-12-27

**Authors:** Sang-Soo Lee, Seil Kim, Hee Min Yoo, Da-Hye Lee, Young-Kyung Bae

**Affiliations:** 1 Bio-Metrology Group, Korea Research Institute of Standards and Science, 267 Gajeong-ro, Yuseong-gu, Daejeon, Korea; 2 Department of Bio-Analytical Science, University of Science & Technology (UST), Daejeon, 34113 Korea; 3 Convergent Research Center for Emerging Virus Infection, Korea Research Institute of Chemical Technology, Daejeon, 34114 Korea; 4 Graduate School of Analytical Science and Technology, Chungnam National University, Daejeon, Republic of Korea

**Keywords:** SARS-CoV-2, Reference material (RM), Digital PCR, Lentiviral particle

## Abstract

Nucleic acid tests to detect the SARS-CoV-2 virus have been performed worldwide since the beginning of the COVID-19 pandemic. For the quality assessment of testing laboratories and the performance evaluation of molecular diagnosis products, reference materials (RMs) are required. In this work, we report the production of a lentiviral SARS-CoV-2 RM containing approximately 12 kilobases of its genome including common diagnostics targets such as *RdRp*, *N*, *E*, and *S* genes. The RM was measured with multiple assays using two different digital PCR platforms. To measure the homogeneity and stability of the lentiviral SARS-CoV-2 RM, reverse transcription droplet digital PCR (RT-ddPCR) was used with in-house duplex assays. The copy number concentration of each target gene in the extracted RNA solution was then converted to that of the RM solution. Their copy number values are measured to be from 1.5 × 10^5^ to 2.0 × 10^5^ copies/mL. The RM has a between-bottle homogeneity of 4.80–8.23% and is stable at 4 °C for 1 week and at −70 °C for 6 months. The lentiviral SARS-CoV-2 RM closely mimics real samples that undergo identical pre-analytical processes for SARS-CoV-2 molecular testing. By offering accurate reference values for the absolute copy number of viral target genes, the developed RM can be used to improve the reliability of SARS-CoV-2 molecular testing.

**Supplementary Information:**

The online version contains supplementary material available at 10.1007/s00216-021-03846-y.

## Introduction

The severe acute respiratory syndrome coronavirus 2 (SARS-CoV-2) that originated in Wuhan, China, in 2019 causes COVID-19 infections [1] and can lead to severe lung damage and mortality [2]. While antigen and antibody testing kits have been developed for the rapid diagnosis of COVID-19, the World Health Organization (WHO) recommends the use of nucleic acid tests (NATs) as a standard means of confirmation of SARS-CoV-2 infection due to their detection capabilities for the unique viral sequences of SARS-CoV-2 [3]. So far, most molecular diagnostic kits use the reverse transcription quantitative polymerase chain reaction (RT-qPCR) method for its high sensitivity and specificity [4, 5].

The importance of reference materials (RMs), meaning homogenous and stable materials with specified properties [6], has been highlighted as molecular diagnostics are widely used in diagnosing infectious diseases worldwide [7]. RMs for specific pathogens are needed for the accurate quantification of their genetic materials by setting reliable values to compare and evaluate the performance of diagnostic kits and to compensate human errors during the process. Current SARS-CoV-2 RMs for molecular diagnosis take various forms, including plasmid DNA, in vitro transcribed RNA [8–12], virus particles containing SARS-CoV-2 RNA [13, 14], and the SARS-CoV-2 virus itself [15]. Comparisons of the established NAT for SARS-CoV-2 have shown that the Cq values from different assays can vary greatly with the same amount of template, indicating the necessity of RMs with specified target concentrations [16, 17]. Various RMs for SARS-CoV-2 are in fact currently available; however, most are limited as users prefer RMs with reference values in absolute quantification of specific genes rather than total nucleic acid measurements or relative quantifications.

Developed by the Korea Research Institute of Standards and Science (KRISS), RM 111–10-507 (batch 2) consists of partial *Orf1ab*, *RdRp*, *N*, *E*, and *S* genes of SARS-CoV-2 enclosed in a lentiviral packaging system. The reference values, or more specifically the copy number concentration of the selected genes, are obtained using one-step reverse transcription droplet digital PCR (RT-ddPCR), which can quantify sequence-specific RNA without extra calibrators [18–21]. The between-bottle homogeneity as well as short-term and long-term stability was tested, and measurement uncertainty was evaluated according to ISO Guide 35 [22].

## Materials and methods

### Cell cultures and preparation of RNA

Jurkat (Korean Cell Line Bank, Seoul, Korea) cells were grown in RPMI 1640 media (Cat. 11,875,093, Gibco, Carlsbad, CA) supplemented with 10% FBS (Cat. 26,140,079, Gibco) at 37 °C, 5% CO_2_. 5 × 10^6^ of these cells were prepared as pellets to extract total RNA using an RNeasy Mini Kit (Cat. 74,104, QIAGEN, Hilden, Germany) according to the manufacturer’s instructions. The extracted RNA was eluted with nuclease-free water (Cat. W4502, Ambion, Austin, TX) and the concentration was measured using a NanoDrop (XP205, METTLER TOLEDO, Columbus, OH). Carrier RNA (Cat. 1,068,337, QIAGEN) and Jurkat cell RNA were used to stabilize the RM.

### Preparation of lentiviral particles

A first-strand cDNA was synthesized from SARS-CoV-2 RNA (NCCP43326) provided by the NCCP (National Culture Collection of Pathogen) using a SuperScript III first-strand synthesis system for RT-PCR (Invitrogen, Waltham, MA) according to the manufacturer’s instructions. Eight target regions were amplified with specific primers (see Electronic Supplementary Material Table S1) and nPfu Forte polymerase (Enzynomics, Daejeon, Korea) or Herculase II Fusion Enzyme with dNTPs Combo (Agilent Technologies, Santa Clara, CA). Through multiple overlap extension PCR [23], insert A was obtained from six PCR products and insert B was obtained from two PCR products. Primers were designed to include sequences with overlapping fragments for PCR stitching as well as extra base pairs including restriction enzyme sites or stop codons. SnaB1, BamH1, and Sal1 restriction enzymes (NEB, Ipswich, MA) were used to insert the final PCR products into the lentiviral vectors (Lugen, Seoul, Korea). Completed *pCDH-A* and *pCDH-B* vectors were verified through Sanger sequencing (Cosmogenetech, Seoul, Korea). Each lentivirus sample (1 × 10^8^ IFU/mL) was obtained through polyethylene glycol (PEG) precipitation using PEG-it Virus precipitation solution (SBI, Palo Alto, CA).

### Production of reference material

Each lentivirus (Lenti-A and Lenti-B) was heat-inactivated for 30 min at 65 °C and diluted together with human RNA into a viral transport medium (Liofilchem, Abruzzo, Italy), which is called a positive RM. For negative RM, only the total RNA from Jurkat cells was spiked into the viral transport medium. The positive and negative RMs were dispensed in 1-mL aliquots into 1.8-mL Cryotubes (Thermo Fisher, Waltham, MA) and stored at − 70 °C.

### RNA extraction, PCR assays, and diagnostic kits

A QIAamp Viral RNA Mini Kit (QIAGEN) was used to extract 60 μL RNA from 140 μL RM according to the manufacturer’s instructions. RNA copy number concentration was measured by one-step RT-ddPCR and one-step RT-qPCR methods using in-house designed assays and published assays by the WHO (Table 1) [3]. In addition, the following commercial diagnostic kits were used: Kaira® 2019-nCoV Detection Kit (Optolane, Seongnam, Korea), PowerChek™ 2019-nCoV Real-time PCR Kit (Kogene, Seoul, Korea), DiaPlexQ™ Novel Coronavirus (2019-nCoV) Detection Kit (Solgent, Daejeon, Korea), and careGENE™ COVID-19 RT-PCR kit (WELLSBIO, Seoul, Korea).

**Table 1:** Sequences of the hydrolysis probes and primers for dPCR and qPCR

Target	Probe sequences	Forward/reverse primers
*RdRp*	5′-[6-FAM]-CCGTAGCTGGTGTCTCTATCTGT-[SFCQ]-3′	5′-TGCAAAGAATAGAGCTCGCA-3′
		5′-CTCCTCTAGTGGCGGCTATT-3′
RdRp*	5′-[6-FAM]-CAGGTGGAACCTCATCAGGAGATGC-[SFCQ]-3′	5′-GTGARATGGTCATGTGTGGCGG-3′
(Germany Charité)		5′-CARATGTTAAASACACTATTAGCATA-3′
*E*	5′-[6-FAM]-TCTTGCTTTCGTGGTATTCTTGCT-[SFCQ]-3′	5′-CGGAAGAGACAGGTACGTTAA-3′
	5′-[HEX]-TCTTGCTTTCGTGGTATTCTTGCT-[SFCQ]-3′	5′-GCAGTAAGGATGGCTAGTGT-3′
E*	5′-[6-FAM]-ACACTAGCCATCCTTACTGCGCTTCG-[SFCQ]-3′	5′-ACAGGTACGTTAATAGTTAATAGCGT-3′
(Germany Charité)		5′-ATATTGCAGCAGTACGCACACA-3′
*N*	5′-[6-FAM]-CACCAATAGCAGTCCAGATGACC-[SFCQ]-3′	5′-ACTCAACATGGCAAGGAAGA-3′
		5′-GCTCTTCGGTAGTAGCCAAT-3′
N*	5′-[6-FAM]-TTGCTGCTGCTTGACAGATT-[SFCQ]-3′	5′-GGGGAACTTCTCCTGCTAGAAT-3′
(China CDC)		5′-CAGACATTTTGCTCTCAAGCTG-3′
N*	5′-[6-FAM]-AGAT/ZEN™/	5′-GACCCCAAAATCAGCGAAAT-3′
(US CDC N1)	ACCCCGCATTACGTTTGGTGGACC-[IBFQ]-3′	5′-TCTGGTTACTGCCAGTTGAATCTG-3′
*S*	5′-[SFC-V]-TCAGACAAATCGCTCCAGGGCA-[SFCQ]-3′	5′-TCTGCTTTACTAATGTCTATGC-3′
		5′-GCTATAACGCAGCCTGTAAA-3′

*FAM*, fluorescein; *HEX*, hexachloro-fluorescein. *Assay published by the WHO [15]

### Reverse transcription droplet digital PCR (RT-ddPCR)

Experiments were conducted with a QX200 Droplet Digital PCR system (Bio-Rad, Hercules, CA). For all comparisons, 5 μL of supermix, 2 μL of reverse transcriptase, 1 μL of 300 mM dithiothreitol (DTT) from a One-Step RT-ddPCR Advanced Kit for Probes (Bio-Rad) with 5 μL of RNA, 2 μL nuclease-free water (Ambion), and standardized primer and probe concentrations of 1 μM forward and reverse primers and 250 nM probe were used. For each assay, a no template control (NTC) reaction was included. An Automated Droplet Generator (Bio-Rad) was used to generate the droplets. PCR was performed in a Veriti 96-well Thermal Cycler (Applied Biosystems, Waltham, MA). The reactions were conducted under the conditions of 60 min at 42 °C, 10 min at 95 °C, 70 cycles of 30 s at 95 °C, 1 min at 59 °C, and 10 min at 98 °C. For the homogeneity test, a reverse transcription temperature of 46 °C was used. After amplification, the plate was loaded onto a QX200 Droplet Reader (Bio-Rad) and analyzed using QuantaSoft software version 1.7.4 (Bio-Rad). All of the thresholds were set up manually to allow the distinction between positive and negative droplets. Only the reactions with more than 10,000 accepted droplets were used for analysis. The final concentration of 1 mL RM was calculated by the following formula, including RNA extraction from the total copy of the PCR results: *copy number concentration of RM* = *total copies in a reaction* / 5 μL × 60 μL / 140 μL × 1000 (copies/1 mL RM). The droplet volume used to calculate copy concentration is 0.85 nL (the manufacturer’s value).

### Reverse transcription digital real-time PCR (RT-drPCR)

Total 30 μL reaction mixtures containing 10 μL 3X Dr. PCR Master mix (Optolane), 10 μL nuclease-free water (Ambion), 5 μL assays with 1 uM of each primer and 0.25 μM FAM probe per reaction, and 5 μL of RNA were used. The reaction mixture was loaded into the cartridge and spread evenly within the chip using a LOAA POSTMAN sample loader (Optolane). The PCR reactions were performed with the LOAA (Optolane) [24]. The reactions were conducted under the conditions of 10 min at 50 °C, 15 min at 95 °C, and 40 cycles of 10 s at 95 °C and 10 s at 57 °C. After amplification, results were analyzed using a Dr. PCR Analyzer version 1.3.24 (Optolane) [24].

### Reverse transcription quantitative PCR (RT-qPCR)

A One Step PrimeScript™ RT-PCR Kit (Takara) with 5 μL of RNA and standardized primers and probe concentrations of 1 μM forward and reverse primers and 0.25 μM probe were used for all comparisons. For each assay, a NTC reaction was included. The PCR cycler conditions were as follows: reverse transcription for 30 min at 42 °C and an initial denaturation for 5 min at 95 °C, followed by 45 cycles of 10 s at 95 °C and 30 s at 59 °C in a StepOne or StepOnePlus Real-Time PCR System (Applied Biosystems). For the commercial SARS-CoV-2 diagnostic kits, RT-qPCR was performed according to the manufacturer’s instructions.

### Homogeneity and stability tests

To determine between-bottle homogeneity, 12 positive tubes of SARS-CoV-2 RM were randomly selected for RT-ddPCR measurements using assays targeting four different genes: *RdRp*, *E*, *N*, and *S.* Between-bottle homogeneity was estimated by subtracting the method repeatability from the between-bottle relative standard deviation (RSD) for each target. Method repeatability for each target was obtained from the RSD between repeated measurements using the same template within an experiment. Short-term stability for shipping, long-term stability, and freeze and thaw tests were performed using up to three positive tubes per experiment with triplicate repeats. In the case of short-term stability, three sets of RMs stored at − 70 °C were randomly selected and transferred to 4 °C and 22 °C. The number of copies was then measured in samples stored for 0, 1, 2, 5, and 7 days. For long-term stability, one or three samples stored at − 70 °C were randomly selected and thawed, and the number of copies was measured at the point of storage at 2, 3, 5, and 6 months. Results were then compared with the results of the homogeneity test. In the freeze and thaw tests, three sets of RMs stored at − 70 °C were melted at 4 °C and then frozen again at − 70 °C 3 or 5 times. Assessment was performed by the following formula [22]:

$$\left|reference\;value-measured\;value\right|\leq k\sqrt{{uncertainty}^2+{uncertainty\;of\;measured\;value}^2}$$

### Uncertainty and statistical analyses

Each source of uncertainty considered was individually evaluated by conducting type A and type B evaluations separately for each target gene [6]. For type A evaluations, standard deviations from independent experiments were calculated. For type B evaluations, the relative standard uncertainty during manual thresholding was calculated as the RSD of three different threshold settings from more than ten independent measurements. In addition, the standard uncertainty from partition volume variations was calculated assuming a uniform rectangular distribution within the range of droplet volumes reported [18, 21, 25–27]. The RSDs of type A and type B were combined by taking the positive square root of the summed squared RSDs to generate a combined relative standard uncertainty. The combined standard uncertainty for each target was combined to generate the expanded uncertainty with a coverage factor of *k* = 2.2 (95% level of confidence, degree of freedom = 11). Experiments were repeated at least in triplicate, or otherwise as indicated in the corresponding figures, and were analyzed with Welch’s *t*-test (two-tailed) using Microsoft Excel 2016 (Microsoft, Redmond, WA). Error bars in the graphical data represent the mean ± standard deviation. Statistical significance was assumed when the *p*-value was lower than 0.05. The plots were drawn using R (4.1.1) with reshape2 (1.4.4), ggplot2 (3.3.5), ggh4x (0.2.0), and readxl (1.3.1) libraries (R Core Team, Vienna, Austria) [28–32].

## Results

### Design and preparation of SARS-CoV-2 reference material

**Fig. 1:**
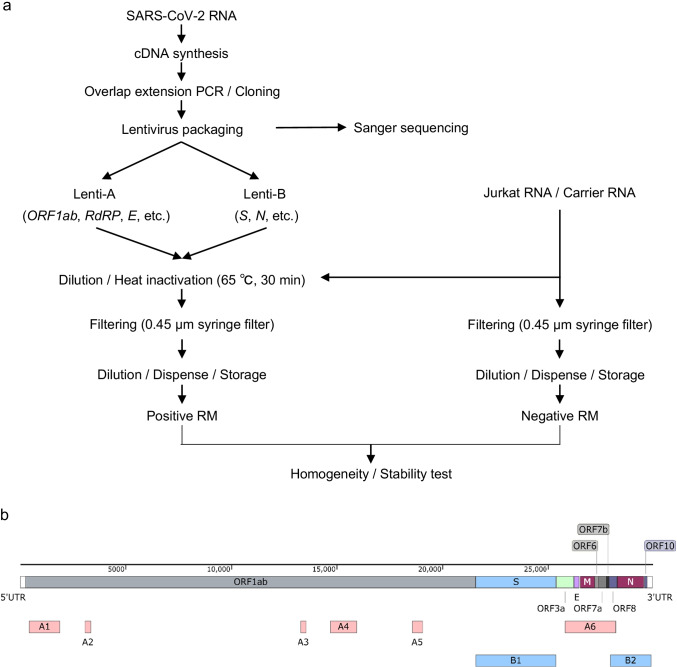
Overall process for the production of the SARS-CoV-2 packaged RNA reference material (RM) and selected target regions. **a** Schematic diagram of the overall procedure for producing and characterizing a positive RM and a negative RM. **b** Schematic presentation of the gene location included in the RM of SARS-CoV-2. A1–A6 are fragments inserted into the *pCDH-A* vector (red boxes), and B1 and B2 are fragments inserted into the *pCDH-B* vector (blue boxes). The features are visualized using a SnapGene 5.3 (GSL Biotech LLC, Chicago, IL)

In this section, the overall scheme for the production of the KRISS 111–10-507 RM (batch 2) is summarized (Fig. 1a). First, cDNA was synthesized from the SARS-CoV-2 total RNA. The selected regions within the viral genome including *ORF1ab*, *E*, *N*, and *S* that are commonly used for COVID-19 diagnosis were individually amplified through PCR (Fig. 1b and Table 2). To improve the biosafety of the lentivirus particles carrying SARS-CoV-2 RNA, a replication-defective lentiviral vector with deleted cytomegalovirus (CMV) promoter sequences was used. In addition, four stop codons were inserted between PCR products (see Electronic Supplementary Material Table S1 and Fig. S1). The summed length of the intended targets was approximately 12 kilobases (kb), which might cause a reduction in the lentivirus packaging efficiency [33]. To ensure a high yield, total six individual PCR products (A1–A6) of *ORF1ab* and *E* were made into fragment A, and two PCR products (B1, B2) of *S* and *N* were made into fragment B (Table 2) using overlap extension PCR [23]. The resulting two fragments (A1–A6 and B1, B2) of approximately 6 kb each were inserted into the lentivirus packaging vector (*pCDH*) to create *pCDH-A* and *pCDH-B*, respectively (see Electronic Supplementary Material Table S1). Some minor variants were found in the RM compared to the reference (GenBank: MW466791) by Sanger sequencing (see Electronic Supplementary Material Fig. S1). The lentiviral particles harvested from *pCDH-A* and *pCDH-B* vector transfection are named Lenti-A and Lenti-B, respectively (Table 2).

**Table 2:** Selected SARS-CoV-2 gene location inserted in the RM

Gene	Protein	Start (bp)	End (bp)		Location inserted in RM (bp)	Fragments	Virus particle
*ORF1ab*	Leader protein	266		805	417–1899	A1	Lenti-A
	Nsp2	806		2719			
	Nsp3	2720		8554	3094–3360	A2	
	RNA-dependent RNA polymerase (RdRp)	13,442		16,236	13,291–13,560	A3	
					14,700–15,950	A4	
	3′-to-5′ exonuclease	18,040		19,620	18,577–19,051	A5	
*S*	Spike glycoprotein	21,563		25,384	21,363–26,001	B1	Lenti-B
*ORF3a*	ORF3a protein	25,393		26,220			
*E*	Envelope protein	26,245		26,472	25,801–28,200	A6	Lenti-A
*M*	Membrane glycoprotein	26,523		27,191			
*ORF8a*	ORF8 protein	27,894		28,259	27,952–29,873	B2	Lenti-B
*N*	Nucleocapsid phosphoprotein	28,274		29,533			
*ORF10*	ORF10 protein	29,558		29,674			

For the positive material, Lenti-A and Lenti-B were filtered after heat inactivation. A small fraction of the concentrated positive materials were used to measure the original concentration. Based on this, we diluted the positive materials down to the value of about 2 × 10^5^ copies/mL for each target in universal transport media supplemented with Jurkat total RNA (2.5 μg/mL) and carrier RNA (2.5 μg/mL). In the case of the negative material, a similar process was applied except for the addition of Lenti-A and Lenti-B particles (Fig. 1a). The KRISS 111–10-507 RM was assembled as a set of positive and negative materials of 1 mL per vial and immediately stored at − 70 °C.

### Determination of the SARS-CoV-2 RM measurement method

**Fig. 2:**
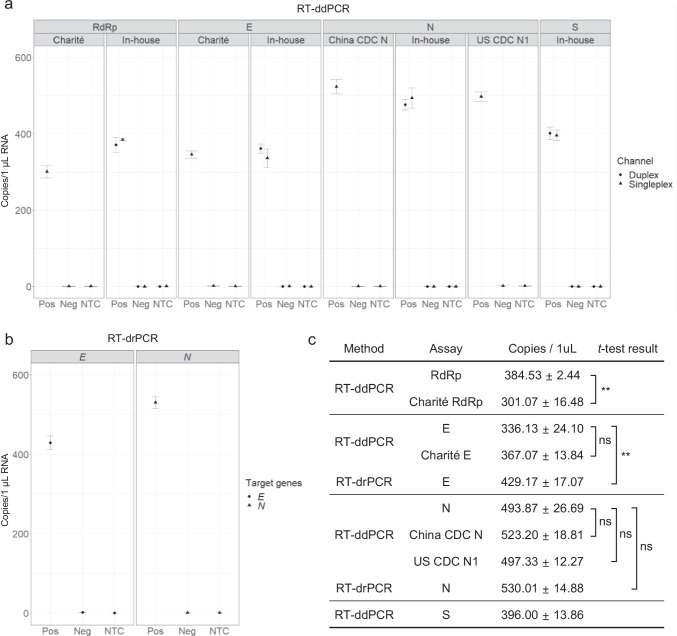
Digital PCR results of RM copy number concentration using assays targeting *RdRp*, *E*, *N*, and *S* genes. **a** Representative reverse transcription droplet digital PCR (RT-ddPCR) results showing the concentration of the positive RM and negative RM using indicated in-house and WHO assays. In the case of the in-house assays, a singleplex assay (triangles) and a duplex assay (circles) are tested and compared. No singleplex or duplex *t*-test results of the same assay are significant. **b** Results of reverse transcription digital real-time PCR (RT-drPCR). Error bars indicate the standard deviation (SD) at each data point with the mean of replicated measurements (*n* ≥ 3). Pos, positive RM; Neg, negative RM; NTC, no template control. **c** Table showing the concentration of 1 μL RNA extracted from the RM and *t*-test results using singleplex data. The asterisks indicate significant differences analyzed by *t*-test (*p < 0.05, **p < 0.01, ***p < 0.001), and ns indicates not significant

To determine the method to measure the copy number concentration of the RM, the RNA extracted from either negative or positive material was used as a template for one-step RT-ddPCR. The analytical performance of in-house designed primer/probe sets (RdRp, E, N, and S) was compared with the widely used WHO assays (Table 1 and Fig. 2a). A series of experiments confirmed that the in-house performance of the assays was acceptable. First, these assays yielded no false-positive results when using RNA from the negative RM or in NTC reactions, showing their high specificity (Fig. 2a). Second, for E and N, the resulting copy number concentrations were comparable to those from the WHO assays (Fig. 2a, c). In the case of RdRp, the in-house assay showed a significantly higher copy number than the Germany Charité RdRp under the same experimental condition (Fig. 2a, c), suggesting a higher analytical sensitivity. The relatively low analytical sensitivity of the Germany Charité RdRp has previously been noticed by RT-qPCR [17, 34]. In addition, the copy numbers using two duplex combinations (RdRp-FAM and S-VIC; N-FAM and E-HEX) were comparable to the respective singleplex results (Fig. 2a). Based on the combined results, the duplex in-house assays using RT-ddPCR were confirmed to be competent for the designed SARS-CoV-2 RM measurements.

To further test the suitability of the RT-ddPCR method, cross-validation was carried out using reverse transcription digital real-time PCR (RT-drPCR), a distinct type of digital PCR (Fig. 2b). The copy number values from the N assays using RT-ddPCR and RT-drPCR were comparable, showing statistically non-significant differences (Fig. 2c). On the other hand, a slightly higher copy number of the *E* gene was obtained using RT-drPCR than using RT-ddPCR (Fig. 2c). In previous and current studies, it is consistently shown that RNA measurement values are similar between RT-drPCR and RT-ddPCR, with some limited exceptions in certain assays [24]. In summary, as the RT-ddPCR method using duplex in-house assays was confirmed to be valid for NAT-based RNA measurements, it was chosen to measure the produced SARS-CoV-2 RM.

### Homogeneity of the SARS-CoV-2 RM

**Fig. 3:**
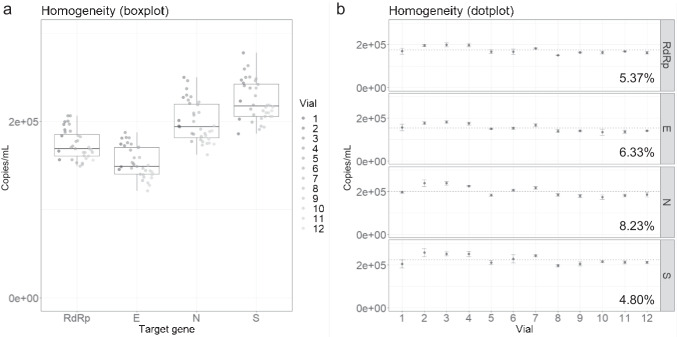
Analysis of the homogeneity test. **a** Boxplot and **b** dotplot showing the homogeneity test results by measuring the copy number concentrations of *RdRp*, *E*, *N*, and *S* genes in 12 vials. **a** The boxplots show the minimum, first quartile (Q1), median, third quartile (Q3), and maximum. **b** Error bars indicate the SD at each data point with the mean of the replicated measurements (*n* = 3). The between-bottle homogeneity values for each gene are indicated in percentage

To test between-bottle homogeneity and determine the reference values of the developed RM, RT-ddPCR was performed using extracted RNA from 12 randomly chosen positive RM vials with three technical replicates. This process ensures that the reference values are valid with any positive vial regardless of the filling order and that the measurement uncertainty covers potential between-bottle variations. The copy number concentrations from the 12 vials were averaged to assign the reference values in 1 mL of positive material. The values for *RdRp*, *E*, *N*, and *S* genes were approximately 1.7 × 10^5^, 1.5 × 10^5^, 2.0 × 10^5^, and 2.2 × 10^5^ copies per mL, respectively (Fig. 3a, b). It is worth noting that these four targets are encapsulated in two lentiviral particles (Lenti-A: *RdRp* and *E*; Lenti-B: *N* and *S*). Consequently, the reference values of the two targets in the same particle are relatively similar. However, these values are not identical probably due to assay-dependent variations [16, 17]. The calculated between-bottle homogeneity of each target was about 4.80–8.23% (Fig. 3c).

### Stability of SARS-CoV-2 RM

**Fig. 4:**
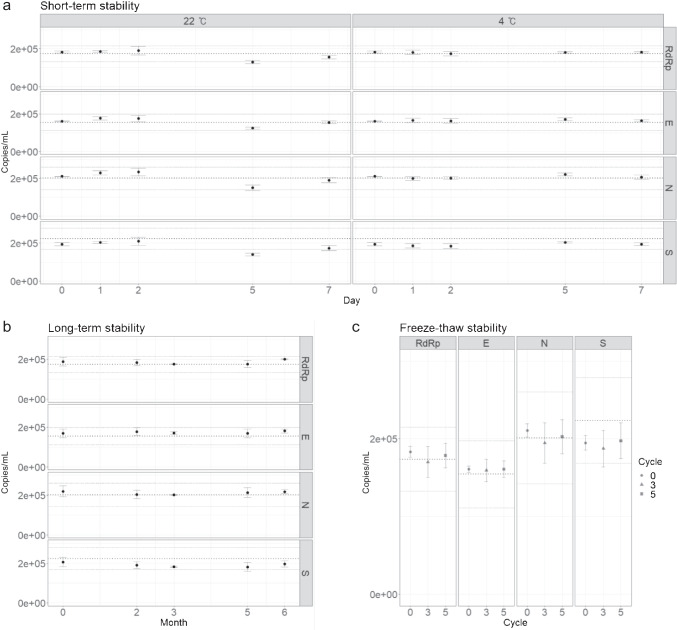
Analysis of the stability tests. The concentrations of the *RdRp*, *E*, *N*, and *S* genes were measured. **a** Graph showing the copy number concentration of the RM stored for the indicated number of days at 4 °C and 22 °C. **b** Graph showing the long-term stability test results for 6 months at − 70 °C. **c** Stability test results following 3 and 5 freeze and thaw cycles. Error bars indicate the SD at each data point with the mean of the replicated measurements (*n* ≥ 3). The thick dotted lines in the middle of the plots indicate the reference values of the RMs, and the upper and lower thin dotted lines indicate the expanded uncertainty of the RMs

A short-term stability study was conducted to test the stability of the reference values over typical transport periods. After positive materials were stored at 4 °C and 22 °C for 0, 1, 2, 5, and 7 days, the number of copies of the *RdRp*, *E*, *N*, and *S* genes was measured. When stored at 4 °C, the RM was found to be stable for 7 days (Fig. 4a). However, the stability of the RM at 22 °C was not as well maintained; storage at 22 °C for 2 days was acceptable, but the copy number values dropped after 5 days, failing the stability assessment tests for all four targets (Fig. 4a). Although the values at 7 days seemed to sufficiently recover to pass the stability tests, it is evident that the overall values had been compromised after storage at 22 °C for 7 days.

Long-term stability tests were also performed to evaluate the RM stability during storage in a specified condition (− 70 °C). Even after 6 months at − 70 °C, the copy numbers did not change significantly (Fig. 4b), passing the stability assessment tests. In addition, as viral particles and RNA are known to be unstable during freeze and thaw cycles, stability assessments during freeze and thaw cycles were conducted. Results confirmed that the RM stability was not affected by up to 5 freeze and thaw cycles (Fig. 4c). These results suggest that the packaged RNA RMs are highly stable at the storage condition and also adequate for transport.

### Effect of variables on measurement uncertainty

To estimate the measurement uncertainty, a number of uncertainty sources were considered. Essentially, the reference values are measured with extracted RNA from twelve different RM vials. This practice was thought to contain multiple uncertainty sources: between- and within-bottle homogeneity, variations in RNA extraction efficiency, variations in reverse transcription efficiency, day-to-day (plate-to-plate) and person-to-person variations, and repeatability of PCR runs.

**Fig. 5:**
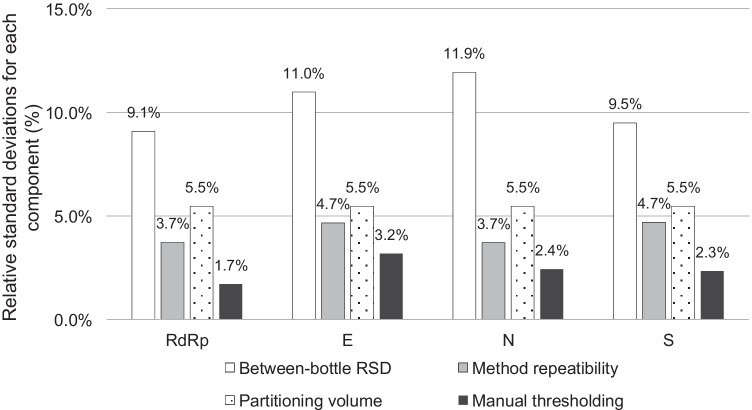
Representative graph showing the uncertainty values. The relative standard deviations (RSD) were calculated per four assays targeting *RdRp*, *E*, *N*, and *S* genes for each of the four components: between-bottle RSD (white), method repeatability (gray), partitioning volume (dotted), and manual thresholding (black)

For type B evaluation, two major components that affect the measurement values during data analysis were considered: uncertainty in assigning the partition volume for the specific dPCR instrument, and uncertainty in manually setting the threshold, especially when there was a group of droplets scattered between the positive and negative droplet population. As the partition volume directly affects the copy number values during dPCR data analysis, the probability distribution was assumed to be rectangular from minimum and maximum reported values [18, 21, 25–27]. This relative standard uncertainty for the partition volume is likely overestimated because these measurements were performed for different PCR supermixes and droplet generators. The relative contributions of the aforementioned sources for each target are compared (Fig. 5). Ultimately, the combined standard uncertainty for each target was expanded with a coverage factor of *k* = 2.20 (95% level of confidence, degree of freedom = 11). The resulting expanded uncertainty and the reference values are summarized in Table 3. By dividing the expanded uncertainty by the reference value for each target, the relative expanded uncertainties are calculated to be 24.7 to 29.4%.

**Table 3:** Reference values of the KRISS RM 111–10-507 batch 2

Target	Reference value (copies/mL)	Homogeneity (%)	Expanded uncertainty (copies/mL)	*k* (95% level of confidence)
*RdRp*	1.7 × 10^5^	5.4	4.1 × 10^4^	2.2
*E*	1.5 × 10^5^	6.3	4.3 × 10^4^	2.2
*N*	2.0 × 10^5^	8.2	5.9 × 10^4^	2.2
*S*	2.2 × 10^5^	4.8	5.5 × 10^4^	2.2

### Measurement of the SARS-CoV-2 RM by RT-qPCR

**Fig. 6:**
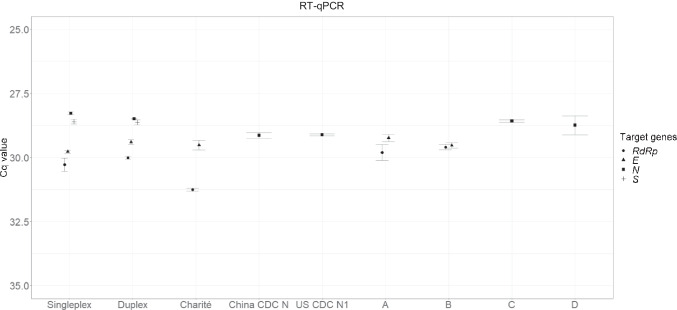
Results of reverse transcription quantitative PCR (RT-qPCR) using diagnostic kits and WHO assays. The graph plots the Cq values of the positive RM from in-house assays, WHO assays, and four commercial diagnostic kits (A–D). The Cq values of the *RdRp* (circles), *E* (triangles), *N* (squares), and *S* (crosses) genes were measured. Error bars indicate the standard deviation (SD) at each data point with the mean of the replicated measurements (*n* ≥ 3)

To provide additional informative values of the KRISS 111–10-507 RM, it was tested via RT-qPCR using in-house and WHO assays as well as commercial diagnostic kits. Similarly as with RT-ddPCR (Fig. 2a), the RT-qPCR results from singleplex and duplex in-house assays were comparable. The Cq values of the four genes (*RdRp*, *E*, *N*, and *S*) ranged from 28 to 30 (Fig. 6). As expected, the in-house assays with the RT-qPCR system performed comparably with Germany Charité RdRp, Germany Charité E, China CDC N, and USA CDC N1 under the same thermocycling and enzyme buffer conditions. Unlike the in-house assay tests, the commercial diagnostic kits, used as instructed, had different assays as well as different enzyme buffer and thermocycling conditions, but their Cq values were comparable (Fig. 6). It is worth noting that the Cq value by Germany Charité RdRp was higher than those by the in-house assay and commercial diagnostic kits, consistent with the previous RT-ddPCR results (Fig. 2a). As a result, the KRISS 111–10-507 RM was found to be compatible with multiple commercial diagnostic kits and WHO assays as well as in-house assays using the RT-qPCR method.

## Discussions

The KRISS 111–10-507 positive RM includes selected regions of SARS-CoV-2 in a lentiviral packaging system. As a reference material, the lentiviral particle has a number of advantages over alternative forms. First, the lentiviral particle form closely mimics real samples from nasal swabs, providing a reference throughout the entire process from RNA extraction to diagnosis. Second, the encapsulated RNAs in lentiviral particles are uniform in length and sequence. The inserted full-length RNAs are transcribed up to the long terminal repeat (LTR) [35, 36]. In contrast, in vitro transcribed RNA, another form of RNA RM, may be heterogeneous in length due to incomplete transcription [37]. In addition, the lentiviral membrane and capsid structure may protect RNA from RNase or hydrolysis [38, 39]. Despite such advantages though, lentiviral particles are considered less safe than naked RNAs or DNA [40, 41]. To circumvent these concerns and improve the biosafety of lentiviral particles carrying SARS-CoV-2 RNA, in addition to heat inactivation of the virus [42], multiple steps were included during the RM production process in this work. A replication-defective lentiviral vector was used, and the CMV promoter in the vector was deleted. Additionally, four extra stop codons in different frames were inserted between PCR products to prevent gene expression. With such measures, the developed lentiviral SARS-CoV-2 RM is a safe and effective material to calibrate and correct bias throughout the entire process of SARS-CoV-2 RNA quantification from RNA extraction to thermocycling, similar to the inactivated SARS-CoV-2 virus RM that does not completely eliminate the possibility of infection. 

The lentiviral SARS-CoV-2 RM provides a specific copy number concentration of the representative targets: 1.5 × 10^5^ to 2 × 10^5^ copies/mL. These RM values in absolute copy number confer a concrete reference across diverse molecular testing applications including RT-qPCR, next-generation sequencing, loop-mediated isothermal amplification, and CRISPR nuclease-based detection [4, 5]. Analyses of the Emergency Use Authorization (EUA) SARS-CoV-2 virus tests in the USA show an unexpectedly broad limit of detection (LoD) of approximately 0.01 to 100 copies/μL [5]. This brings up a critical issue: for every tenfold increase in LoD, the false-negative rate is expected to increase by 13% [42]. This can be interpreted to derive from the absence of reliable RMs that are internationally available, currently unsatisfactory method validation, or simple inter-laboratory and inter-personnel variations. In this light, the KRISS 111–10-507 RM can be broadly applied to increase reliability in molecular testing, such as in comparisons of diverse NAT methods or EUA diagnostic kits, the determination of a valid LoD for a given method, the validation of a new analytical process, and evaluations of an instrument or personnel. In particular, the reference values in copy number units can serve as a firm standard to compare different RT-qPCR based methods, represented in Cq values.

The major measurement uncertainty of the developed RM stems from pre-analytical processes such as RNA extraction and the handling of viscous solutions. After combining the considered uncertainty values, the expanded uncertainty is summarized in Table 3. These values were obtained using RNA extracted from a subsample of the RM with a specific commercial viral RNA extraction kit; it has been shown that RNA extraction efficiency varies widely depending on the method applied and the personnel [43, 44]. Another source of variation in the reference values is the viscosity of the viral transport medium (VTM), which is used as the matrix of this RM for its protective and stabilizing properties. However, its high viscosity can interfere with an even dispersion of the virus and reduce the precision of pipetting during subsampling. It is supposed that the VTM matrix contributed to the lower homogeneity of the lentivirus-based SARS-CoV-2 RM compared to the in vitro transcribed RNA RM [12].

The KRISS 111–10-507 RM as a set of positive and negative samples is widely applicable. The positive RM can substitute for real patient samples of confirmed cases, especially in the beginning of a pandemic or if the biosafety level of the related facilities is not suitable. Together with the positive samples, the negative RM containing only human RNA can be used as a practice material to improve proficiency or as a test material in external and internal quality assessments in testing laboratories. In summary, the KRISS 111–10-507 RM provides valuable measurement standards for SARS-CoV-2 molecular testing.

## Supplementary Information

Below is the link to the electronic supplementary material.

Supplementary file1 (DOCX 146 kb)
