# Supplementary material for: Development of SARS-CoV-2 packaged RNA reference material for nucleic acid testing
Source: Anal Bioanal Chem. 2021 Dec 27;414(5):1773–85. doi: 10.1007/s00216-021-03846-y (PMC8711077; doi:10.1007/s00216-021-03846-y)
Supplement: Supplementary file 1 — Supplementary file1 (DOCX 146 kb) [file 216_2021_3846_MOESM1_ESM.docx]

**Supplementary Information:**

**Development of SARS-CoV-2 Packaged RNA Reference Material for nucleic acid testing**

**Sang-Soo Lee^1^, Seil Kim^1,2,3^, Hee Min Yoo^1,2^, Da-Hye Lee^1*^, and Young-Kyung Bae^1*^**

^1^ Bio-Metrology Group, Korea Research Institute of Standards and Science, Daejeon, Korea

^2^ Department of Bio-Analytical Science, University of Science & Technology (UST), Daejeon 34113, Korea

^3^ Convergent Research Center for Emerging Virus Infection, Korea Research Institute of Chemical Technology, Daejeon 34114, Korea

^*^Corresponding authors: Da-Hye Lee, and Young-Kyung Bae, Bio-Metrology Group, Korea Research Institute of Standards and Science, 267 Gajeong-ro, Yuseong-gu, Daejeon, Korea. E-mail address: [dahye.lee04@kriss.re.kr](mailto:dahye.lee04@kriss.re.kr), ybae@kriss.re.kr

| Target | # of parts | Location (bp) | Forward/Reverse primers | Structures of primers |
| --- | --- | --- | --- | --- |
| *ORF1* | #1 | 417-1899 | 5’-TCGAGCTCAAGCTTCGAATTCTACGT  AGTGGCTTAGTAGAAGTTGAAAAAGG-3’ | 5’-Vector-EcoR1-SnaB1-#1-3’ |
|  |  |  | 5’-GTTGATTCTATCGTACAACACGAGCAGCC-3’ | 5’-#2-5 bp (stop codon)-#1-3’ |
|  | #2 | 3094-3360 | 5’-TGTACGATAGAATCAACTCAATATGAGTATGGTACTGAA-3’ | 5’-#1-5 bp (stop codon)-#2-3’ |
|  |  |  | 5’-TCACAATTACTAAATAACCAC  TAAAACTATTCACTTCAATAGT-3’ | 5’-#3-5 bp (stop codon)-#2-3’ |
| *RdRp* | #3 | 13291-13560 | 5’-TTATTTAGTAATTGTGACTTAA  AAGGTAAGTATGTACAAATAC-3’ | 5’-#2-5 bp (stop codon)-#3-3’ |
|  |  |  | 5’-AAGTCACACTAATCATTGTAGATGTCAAAAGCCCT-3’ | 5’-#4-5 bp (stop codon)-#3-3’ |
|  | #4 | 14700-15950 | 5’-AATGATTAGTGTGACTTTGCTGTGTCTAAGGG-3’ | 5’-#3-5 bp (stop codon)-#4-3’ |
|  |  |  | 5’-ACCATGACCGGTGGATCCAT  TCTTGATGGATCTGGGTAAGG-3’ | 5’-Vector-BamH1-#4-3’ |
|  | #5 | 18577-19051 | 5’-TCGAGCTCAAGCTTCGAATTCGGAT  CCAGAGTCGTATTTGTCTTATGGGC-3’ | 5’-Vector-EcoR1-BamH1-#5-3’ |
|  |  |  | 5’-TGGGTTTCATCTAGCTTTAGGGTTACCAATGTCG-3’ | 5’-#6-5 bp (stop codon)-#5-3’ |
| *E* | #6 | 25801-28200 | 5’-AAGCTAGATGAAACCCATTACTTTATGATGCCAACT-3’ | 5’-#5-5 bp (stop codon)-#6-3’ |
|  |  |  | 5’-ACCATGACCGGTGGATCCGTCGACT  CATTAACAACGCACTACAAGACTACC-3’ | 5’-Vector-BamH1-Sal1-  5 bp (stop codon)-#6-3’ |
| *S* | #7 | 21363-26001 | 5’-CGTACGTATCCAATTCAGTTGTCTTCCTATTCTT-3’ | 5’-SnaB1-#7-3’ |
|  |  |  | 5’-ACTGTAAACTAC TAATACAACACAGTCTTTTACTCCAG-3’ | 5’-#8-#7-3’ |
| *N* | #8 | 27952-29873 | 5’-GACTGTGTTGTATTAGTAGTTTACAGTCATGTACTC-3’ | 5’-#7-#8-3’ |
|  |  |  | 5’- CGGTCGACTTTGTCATTCTCCTAAGAAGCTATTAAAAT-3’ | 5’-Sal1-#8-3’ |
| bp, base pair; EcoR1,SnaB1, Sal1, each restriction enzyme site sequence; #1 - #8, about 10 - 20 bp sequences of each target; 5 bp, extra sequences including stop codon sequence; Vector, *pCDH* vector sequence. | | | | |

**Table S1.** Sequences of the primers for PCR stitching and cloning

**Fig. S1**


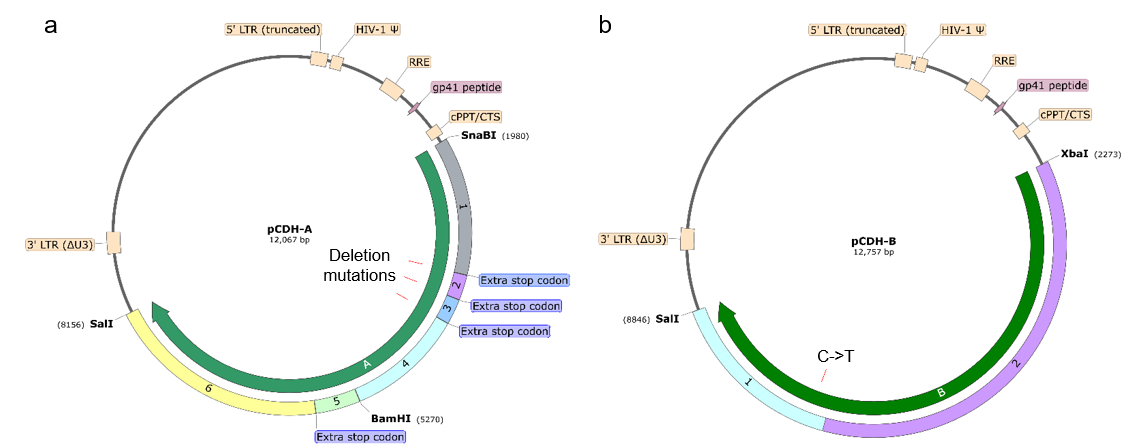


**Fig. S1** Vector maps for lentivirus packaging. Maps of the **a** *pCDH-A* vector into which the A1–A6 fragments were inserted and the **b** *pCDH-B* vector into which the B1 and B2 fragments were inserted, including point mutation and deletions confirmed through Sanger sequencing. Extra base pairs including a stop codon were inserted between the A1–A6 fragments. Restriction enzyme sites used for cloning are specified. Features are visualized using a SnapGene 5.3 (GSL Biotech LLC).
